# Biomechanical Material Characterization of Stanford Type-B Dissected Porcine Aortas

**DOI:** 10.3389/fphys.2018.01317

**Published:** 2018-09-26

**Authors:** Aashish Ahuja, Jillian N. Noblet, Tony Trudnowski, Bhavesh Patel, Joshua F. Krieger, Sean Chambers, Ghassan S. Kassab

**Affiliations:** ^1^Cardiovascular Mechanics and Diseases, California Medical Innovations Institute, San Diego, CA, United States; ^2^Cook Medical Inc., Bloomington, IN, United States

**Keywords:** aortic dissection, material behavior, Holzapfel–Gasser–Ogden, planar biaxial testing, layered model

## Abstract

Aortic dissection (AD) involves tearing of the medial layer, creating a blood-filled channel called false lumen (FL). To treat dissections, clinicians are using endovascular therapy using stent grafts to seal the FL. This procedure has been successful in reducing mortality but has failed in completely re-attaching the torn intimal layer. The use of computational analysis can predict the radial forces needed to devise stents that can treat ADs. To quantify the hyperelastic material behavior for therapy development, we harvested FL wall, true lumen (TL) wall, and intimal flap from the middle and distal part of five dissected aortas. Planar biaxial testing using multiple stretch protocols were conducted on tissue samples to quantify their deformation behavior. A novel non-linear regression model was used to fit data against Holzapfel–Gasser–Ogden hyperelastic strain energy function. The fitting analysis correlated the behavior of the FL and TL walls and the intimal flap to the stiffness observed during tensile loading. It was hypothesized that there is a variability in the stresses generated during loading among tissue specimens derived from different regions of the dissected aorta and hence, one should use region-specific material models when simulating type-B AD. From the data on material behavior analysis, the variability in the tissue specimens harvested from pigs was tabulated using stress and coefficient of variation (CV). The material response curves also compared the changes in compliance observed in the FL wall, TL wall, and intimal flap for middle and distal regions of the dissection. It was observed that for small stretch ratios, all the tissue specimens behaved isotropically with overlapping stress–stretch curves in both circumferential and axial directions. As the stretch ratios increased, we observed that most tissue specimens displayed different structural behaviors in axial and circumferential directions. This observation was very apparent in tissue specimens from mid FL region, less apparent in mid TL, distal FL, and distal flap tissues and least noticeable in tissue specimens harvested from mid flap. Lastly, using mixed model ANOVAS, it was concluded that there were significant differences between mid and distal regions along axial direction which were absent in the circumferential direction.

## Introduction

Aortic dissection (AD) is the most common life-threatening disorder affecting the aorta (Hagan et al., 2000). AD is classified as Stanford type-B if it originates distal to the left subclavian artery and does not involve ascending aorta. In type-B dissection, there is separation and propagation between the intima–media where blood enters the layers of the aortic wall to create a false channel, known as the false lumen (FL) in addition to the normal endothelialized channel referred to as the true lumen (TL). The layer of the aorta dissected from its wall is called the intimal flap. The primary pathological changes in the aortic wall leading to AD is attributed to two major theories (Mann et al., 2015). The first ascribes primacy to the development of an intimal tear, followed by penetration of blood from the aortic lumen into a weakened, susceptible medial space (characterized by elastic degeneration of the vessel). The second hypothesis is that initial rupture of the vasa vasorum leads to hemorrhage within the aortic wall and subsequent intimal disruption and propagation of a dissection flap. The dynamics of the intimal flap and the dilation of the FL during the cardiac cycle can cause malperfusion of the vital organs (usually kidneys) and can lead to adverse life-threatening events. AD has been linked with clinical complications such as aneurysmal formation, aortic wall rupture, aortic wall regurgitation, pericardial effusion causing tamponade, hypotension/shock, and malperfusion syndromes leading to end organ ischemia (Erbel et al., 2001; Greenberg et al., 2003; Nienaber and Eagle, 2003; Golledge and Eagle, 2008; Juang et al., 2008; Patel et al., 2014). The two most important acquired risk factors related to the development of AD include hypertension and atherosclerosis. Hypertension has been linked with Stanford Type B dissections in 70% of cases. This is almost twice as many as the number of incidences with type A dissections where hypertension was found to be the leading cause (36%; Hagan et al., 2000). The propensity to AD is also amplified due to genetic diseases and connective tissue disorders. Syndromes such Marfan, Ehlers-Danlos, Loeys-Dietz, familial AD, and annulo-aortic ectasia are all implicated in the development of thoracic aortic aneurysm and dissection (Halme et al., 1985).

The incidence of AD in the United States is approximately 2,000 cases per year and early mortality is as high as 1% per hour if untreated (Vecht et al., 1980; Roberts, 1981). Currently, there are three modes of treating patients suffering from AD: medical management, open surgery, and endovascular treatment. While medical management is suggested for patients that have uncomplicated dissections, for complicated dissections, open surgery or endovascular grafting is recommended. The design and use of endovascular grafts or bare metal stents can provide sufficient radial forces on the intimal flap to push it back against the FL wall and allow reconstitution of the aorta without imposing high mechanical stresses on the FL wall. The research and development of effective mechanical devices for endovascular grafting would require the use of computational techniques to analyze the structural interaction between the rigid stents (usually composed of Stainless steel or Nitinol alloy) and different tissue segments of the dissected aorta (i.e., Intimal flap, FL wall, and TL wall). Unfortunately, the “building elements” for computational model such as a suitable constitutive model that characterizes the mechanical behavior of a dissected aorta by providing a mathematical formulation for the stress–strain relation is currently lacking (Babu et al., 2015). Structural continuum constitutive models of the different layers of aorta integrate information about the tissue morphology and therefore assess the interrelation between the structure and response to mechanical loading. Fiber-reinforced structural models of different layers of aorta, namely media and adventitia, have been presented in Holzapfel et al. (2000) and Holzapfel and Gasser (2001), but material characterization of intima–media and media–adventitia layers from porcine aortas suffering from dissection is not available. The current Finite Element Analysis (FEA) and Fluid–Structure Interaction models for dissected aorta assume linear elasticity or simplified hyperelasticity for different regions of the dissected aorta (Alimohammadi et al., 2015). The goal of this paper was to develop a novel non-linear regression material model using data from planar biaxial testing on dissected porcine aortas and empirically fit it to a five parameter form of Holzapfel–Gasser–Ogden hyperelastic strain energy function (Gasser et al., 2006). It was also hypothesized that there was a variability in the stresses generated due to loading among tissue specimens derived from different regions of the dissected aorta and hence, one should use region-specific material models when simulating type-B AD. To test the hypothesis, the variability in the tissue specimens harvested from (*n* = 5) pigs was tabulated using stress as the variable and coefficient of variation (CV) as the statistical method. Also, the analysis compared the changes in the compliance and regional variability observed in the TL wall, FL wall, and intimal flap harvested from middle and distal regions of the dissection. The passive behavior was the focus of this work, while the active response will be studied in a subsequent work once the passive foundation is established here. It is hopeful that the biomechanical characterization of a layered model for dissected aorta will expedite the development of endovascular therapy for successfully sealing the FL thereby reducing mortality and future reinterventions.

## Materials and methods

### Materials

The data for the material behavior was collected from five porcine aortas obtained from a slaughterhouse. The aortas were obtained from ~100 kg swine that had been raised on a farm (Sierra for medical sciences, Whittier, CA, USA). The descending thoracic part of the aortas was harvested, cleaned, and flushed with 0.9% NaCl physiological saline solution and later stored in saline at 4°C to slow down any enzymatic tissue breakdown (Rashid et al., 2013). The mechanical testing of the samples were completed within 16 h of tissue harvest.

### Dissection

**Figure 1:**
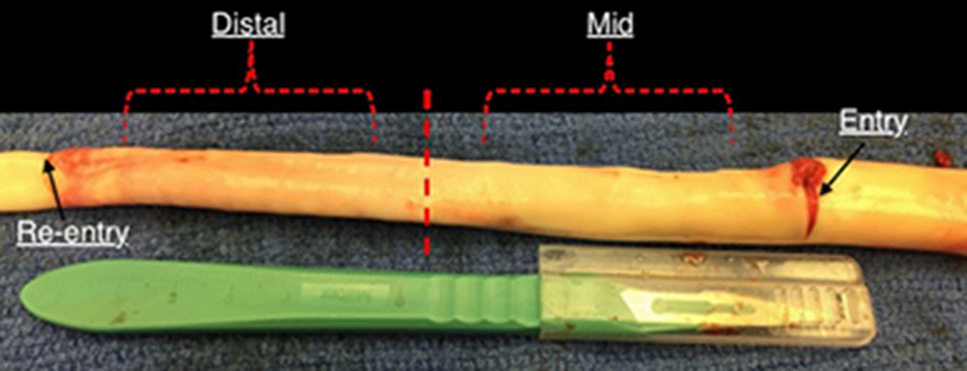
An inverted aorta with dissection. An entry was initially created carefully in the descending thoracic aorta and propagated using forceps to the distal region of the aorta where a pocket of re-entry is created. Tissue specimens from two regions (mid and distal) are extracted and tested on planar biaxial testing machine for material characterization.

A healthy porcine aorta was inverted exposing the intima and dissections were created ~5–6 cm from the vessel start (~6–8 cm from the left subclavian artery). Dissections in healthy descending thoracic aorta represented the case of acute Type-B AD. The percent circumferential length of the entry tear was calculated as 100X (perimeter of the flap/circumference of the vessel). The perimeter of the flap was calculated by measuring the average length of the two edges of the flap in an ultrasound image representing cross-section of the entry tear (Peelukhana et al., 2016; Canchi et al., 2017). Using a surgical blade, a cut was made in the inner lining of the vessel. The layers were separated using the surgical blade and advanced using a fine-tip forceps to the desired axial length. A resulting intimal flap of about ~10–13 cm in length was created due to surgical dissection as shown in Figure 1. At the end of dissection, a reentry was created and the flap separated the TL from the FL in the vessel.

### Mechanical experiments on dissected tissues using displacement controlled biaxial protocols

**Figure 2:**
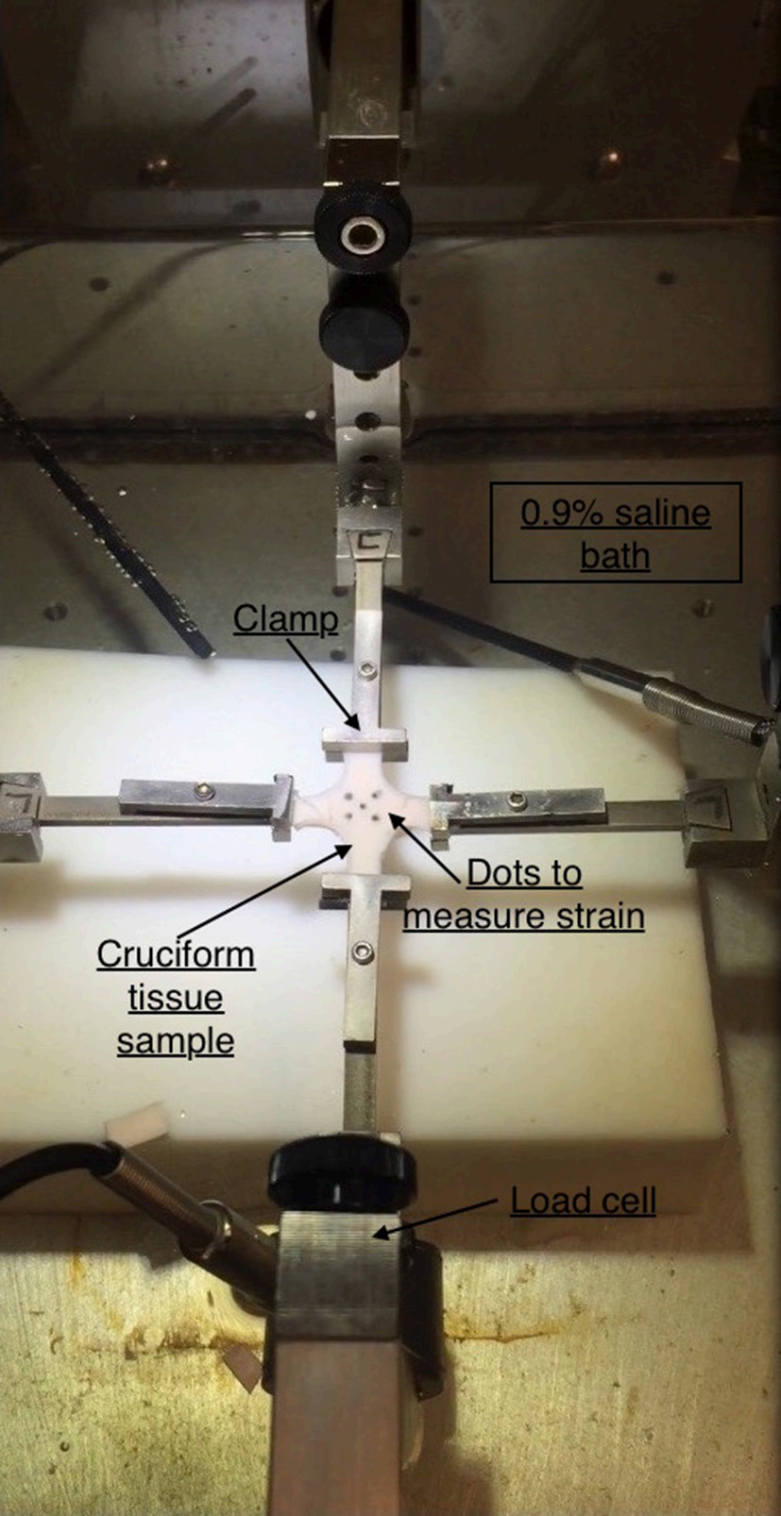
Planar biaxial testing setup. A cruciform specimen is suspended using clamps which is stretched along x- and y-axes. The x-axis represents the circumferential direction while y-axis represents the axial direction.

The planar mechanical biaxial experiments were performed using a custom-built planar biaxial testing machine shown in Figure 2. The instrument consisted of four motors with attached encoders and each motor had a maximum displacement of 12 mm. The force on the tissue was measured using 1,000-g submersible load cells installed in both x- and y-directions. The strain was measured with the “Bose® digital video extensometer,” and the entire system was controlled and monitored using WinTest® version 7 software. The extensometer had a sampling rate of up to 200 Hz. The experiments were conducted at a sampling rate of 0.02 Hz to achieve quasi-static loading conditions. Tissue specimens were oriented in the circumferential and longitudinal directions and attached to the linear arms using clamps. The specimens were immersed in 0.9% NaCl physiological saline solution maintained at 37°C. Several previous studies such as Rassoli et al. (2014), Zemánek et al. (2009), and Jhun et al. (2009) had also used 0.9% saline solution for biaxial testing on soft tissue and bioartificial tissue specimens but did not report any deteriorating effects on their structural integrity. Each specimen was cut into a cruciform shape of 15 × 15 mm cross-sectional area such that the arm width, *w*, was 5 mm. Four graphite markers were applied to the central region (away from corners and arms to avoid errors due to end effects) of the cruciform specimen and the marker positions during deformation were recorded. Using a dedicated proprietary software (prepackaged with Bose® digital video extensometer), the displacements of the four markers were tracked and the recorded data was used to calculate the circumferential (ϵ_θ_) and longitudinal (ϵ_*z*_) strains in the tissue specimen.

The mechanical testing showed that preconditioning of 10 loading–unloading cycles on specimens could eliminate the viscoelastic response and provide reproducible curves. An additional 10 cycles were done to ensure there was no load cell drift during mechanical testing. For each specimen, enzymatic degradation was not induced as the tissue was tested within 16 h (Rashid et al., 2013). The strains and loads along the two axes were recorded for each of the five different displacement protocols (1:1^1^, 1.5:1, 2:1, 1:1.5, 1:2). After completing the preconditioning and reproducibility of force-displacement curves, the loading curves from succeeding three cycles were chosen for the determination of material parameters.

The Cauchy stress was computed for both circumferential and longitudinal directions. It is defined as:

(1) $$\begin{array}{ccc} \sigma_{\theta \theta } & = & \left(\frac{F_{\theta }}{tw}\right)\lambda_{\theta } \end{array}$$

(2) $$\begin{array}{rcl} \sigma_{zz} & = & \left(\frac{F_{z}}{tw}\right)\lambda_{z} \end{array}$$

where stresses along circumferential and longitudinal directions were given as σ_θθ_ and σ_*zz*_, respectively. *F*_θ_ and *F*_*z*_ were loads registered by the load cells of ElectroForce TestBench instrument along the two directions. The variables *t* and *w* (= 5 mm) were the initial thickness and width of the tissue sample, respectively and λ_θ_ (= ϵ_θ_+1) and λ_*z*_ (= ϵ_*z*_+1) were the stretches in circumferential and longitudinal directions which were measured using the CCD camera mounted over the specimen. The thickness of the sample was measured using a Mitutoyo Absolute Digimatic caliper-type micrometer. For each specimen, thickness was measured at four locations using the micrometer. The average measurement for each specimen was recorded and provided in Table 1. The shear strains were measured by the data acquisition system and were small and not accounted for in the constitutive model.

**Table 1:** Specifications of tissue sample and test protocols used for its material characterization.

**Pig number**	**Region of thoracic aorta and displacement-controlled protocols**
Pig 1	Mid TL wall (1:1),
	Mid Flap (1:1, 1:1.5, 1:2, 1.5:1, 2:1),
	Mid FL wall (1:1, 1:1.5, 1:2, 1.5:1, 2:1),
	Distal FL wall (1:1, 1.5:1, 2:1),
	Distal Flap (1:1, 1:1.5, 1:2, 1.5:1, 2:1)
Pig 2	Mid TL wall (1:1, 1:1.5, 1:2, 1.5:1, 2:1),
	Mid Flap (1:1, 1:1.5, 1:2, 1.5:1, 2:1),
	Mid FL wall (1:1, 1:1.5, 1:2, 1.5:1, 2:1),
	Distal FL wall (1:1, 1:2, 1.5:1, 2:1),
	Distal Flap (1:1, 1:1.5, 1:2, 1.5:1, 2:1)
Pig 3	Mid TL wall (1:1, 1:1.5, 1:2, 1.5:1, 2:1),
	Mid Flap (1:1, 1:1.5, 1:2),
	Mid FL wall (1:1, 1:1.5, 1:2, 1.5:1, 2:1),
	Distal FL wall (1:1),
	Distal Flap (1:1, 1:1.5, 1:2, 1.5:1, 2:1)
Pig 4	Mid TL wall (1:1, 1:1.5, 1:2, 1.5:1, 2:1),
	Mid Flap (1:1, 1:1.5, 1:2),
	Mid FL wall (1:1),
	Distal FL wall (1:1, 1:1.5, 1:2, 1.5:1, 2:1),
	Distal Flap (1:1, 1:1.5, 1:2, 1.5:1, 2:1)
Pig 5	Mid TL wall (1:1, 1:1.5, 1:2, 1.5:1),
	Mid Flap (1:1),
	Mid FL wall (1:1),
	Distal FL wall (1:1, 1.5:1, 2:1),
	Distal Flap (1:1, 1:1.5, 1:2, 1.5:1, 2:1)

#### Theory

Using the displacements recorded on the planar biaxial tests, we computed the Green strains (E) in the principal material directions. Strains were represented in terms of the in-plane deformation gradient tensor, F, as:

(3) $$\begin{array}{r} \text{E}=\frac{1}{2}({\text{F}}^{\text{T}}\text{F}-\text{I}) \end{array}$$

where I was the identity tensor and F^T^ was the transpose of deformation gradient tensor F. The strain-energy function (SEF) proposed on Gasser et al. (2006) was used to represent the inherent hyperelasticity of the aortic tissue. It was given as an additive split of the isochoric SEF into a part associated with isotropic deformations and a part associated with the anisotropic deformations as given by:

(4) $$\begin{array}{r} \text{Ψ}={\text{Ψ}}_{iso}+{\text{Ψ}}_{aniso} \end{array}$$

The isotropic component (Ψ_*iso*_) was associated with the mechanical response of elastin and smooth muscle cells in the passive state (Gundiah et al., 2009) and was described as:

(5) $$\begin{array}{r} {\text{Ψ}}_{iso}=C_{10}\left(I_{1}-3\right) \end{array}$$

where *C*_10_ was a material constant and *I*_1_ represented the first invariant of the Cauchy-Green tensor (Spencer, 1971). The anisotropic component (Ψ_*aniso*_) was related to the response of collagen fibers to loading of the tissue specimen. The collagen fibers were crimped at low stretches of the tissue and are not involved in its extension. At higher stretches, the fibers were elongated and were responsible in reinforcing the tissues. An exponential function was used to describe the strain energy stored in the collagen fibers:

$$\begin{array}{rcl} {\text{Ψ}}_{aniso} & = & \frac{k_{1}}{2k_{2}}\left(\exp\left\{k_{2}{\left[\kappa I_{1}+\left(1-3\kappa \right)I_{4}-1\right]}^{2}\right\}\right) \end{array}$$

(6) $$\begin{array}{ll} + & \frac{k_{3}}{2k_{4}}\left(\exp\left\{k_{4}{\left[\kappa I_{1}+\left(1-3\kappa \right)I_{6}-1\right]}^{2}\right\}\right) \end{array}$$

where *I*_4_, *I*_6_≥1 characterized the mechanical response in the preferential directions of the fibers. *k*_1_>0 and *k*_3_>0 were stress like parameters while *k*_2_>0 and *k*_4_>0 were dimensionless. The parameter κ∈[0, 1/3] was also dimensionless and accounted for fiber dispersion. The preferred directions for the fibers contributing to the SEF was represented by invariants *I*_4_ *and I*_6_. The anisotropy directions in tissues were assumed to be helically oriented at ±θ degrees with respect to the longitudinal direction (Holzapfel et al., 2000). Therefore, invariants *I*_4_ *and I*_6_ became equal and were given as:

(7) $$\begin{array}{c} I_{4},\;I_{6}=\lambda_{\theta }^{2}{\text{cos}}^{\text{2}}\theta +\lambda_{z}^{2}{\text{sin}}^{\text{2}}\theta \end{array}$$

A value of κ close to 0 indicated concentration of the fibers along the preferred orientation θ while a value closer to 1/3 suggested dispersion of the fibers. Also, since each family of fibers represented the main direction we assumed same mechanical response along θ degrees, therefore, *k*_1_ = *k*_3_ and *k*_2_ = *k*_4_. The value of Ψ_*aniso*_ was only valid when the tissue was stretched and became zero when *I*_4_, *I*_6_ < 1.

The vascular wall layers, namely TL wall, intimal flap, and FL wall, were incompressible. This meant that the volume of these tissue specimens remained conserved after deformation. As a result, the Jacobian of the deformation gradient, represented as *J* = det(*F*) and defined as the product of stretches in the principal directions, λ_θ_λ_*z*_λ_*r*_ was equal to 1. The vessel wall layers were regarded to be composed of elastin, smooth muscle cells and collagen fibers. In a planar biaxial testing experiment of tissue specimen with the axes aligned with the longitudinal and circumferential directions, the deformation gradient, *F* and corresponding Cauchy stress tensor, σ were given as:

(8) $$F=[\begin{array}{ccc} \lambda_{\theta } & 0 & 0\\ 0 & \lambda_{z} & 0\\ 0 & 0 & 1/\lambda_{\theta }\lambda_{z}\; \end{array}],\sigma =[\begin{array}{c} \sigma_{\theta \theta }\\ \sigma_{zz}\\ 0 \end{array}]=[\begin{array}{c} \lambda_{\theta }\frac{\partial \Psi }{\partial \lambda_{\theta }}\\ \lambda_{z}\frac{\partial \Psi }{\partial \lambda_{z}}\\ 0 \end{array}]$$

The values of stresses along circumferential and axial directions were obtained using Equation (9):

(9) $$\begin{array}{l} \sigma_{\theta \theta }=\;\lambda_{\theta }[\;2C_{10}\;\left(\lambda_{\theta }-\frac{1}{\lambda_{\theta }^{3}\lambda_{z}^{2}}\right)+4k_{1}\left(\kappa I_{1}+\left(1-3\kappa \right)I_{4}-1\right)\\ e^{\left\{k_{2}{\left(\kappa I_{1}+\left(1-3\kappa \right)I_{4}-1\right)}^{2}\right\}}\left(\kappa \left(\;\lambda_{\theta }-\frac{1}{\lambda_{\theta }^{3}\lambda_{z}^{2}}\;\right)+\lambda_{\theta }\left(1-3\kappa \right)cos^{2}\alpha )\;\right)]\\ \sigma_{zz}=\;\lambda_{z}[\;2C_{10}\;\left(\lambda_{z}-\frac{1}{\lambda_{\theta }^{2}\lambda_{z}^{3}}\right)+4k_{1}\left(\kappa I_{1}+\left(1-3\kappa \right)I_{4}-1\right)\\ e^{\left\{k_{2}{\left(\kappa I_{1}+\left(1-3\kappa \right)I_{4}-1\right)}^{2}\right\}}\left(\kappa \left(\;\lambda_{z}-\frac{1}{\lambda_{\theta }^{2}\lambda_{z}^{3}}\;\right)+\lambda_{z}\left(1-3\kappa \right)cos^{2}\alpha )\;\right)] \end{array}$$

#### Statistical methods

The coefficient of determination *R*^2^∈[0, 1] and the root square of the reduced chi-square ε∈[0, 1] were used as a measure of correlation between the model-derived values and the experimental data. They were defined as:

(10) $$\begin{array}{rcl} R^{2}\left(A\right) & = & \frac{\sum_{f=zz,\theta \theta }\sum_{q=1}^{n}{\left(A_{q,f}^{m}-A_{q,f}^{exp}\right)}^{2}}{\sum_{f=zz,\theta \theta }\sum_{q=1}^{n}{\left(A_{avg,f}^{exp}-A_{q,f}^{exp}\right)}^{2}} \end{array}$$

(11) $$\begin{array}{rcl} \varepsilon \left(A\right) & = & \sqrt{\frac{1}{n-n_{v}}\underset{f=zz,\theta \theta }{\sum }\overset{n}{\underset{q=1}{\sum }}{\left(\frac{A_{q,f}^{m}-A_{q,f}^{exp}}{A_{avg,f}^{exp}}\right)}^{2}} \end{array}$$

where *A* = σ_*zz*_, σ_θθ_, the subscript “*avg*” indicated the average of the experimental values over all *n* data points, and *n*_*v*_ = 5 referred to the number of unknown parameters for the model. A high value of *R*^2^ indicated that a good fit was globally obtained. A low value of ε revealed that the differences between model predicted and experimental values were not significant for each data point. The model was fitted to all protocols. Fitting was considered acceptable for *R*^2^>0.8 and ε < 0.25 over all data points, and *R*^2^>0.9 with ε < 0.2 for data from equibiaxial protocol. A particular importance was given to data from equibiaxial protocol since equibiaxial displacement conditions were typically favored for model fitting and, in some cases, only data from equibiaxial conditions were retained. This was justified by the fact that, generally, smoother deformation data was captured under equibiaxial conditions. Regardless, we still considered other protocols to inform the model with more data about the material, albeit a particular focus was given to the equibiaxial-displacement data.

A statistically independent mixed model ANOVAS were conducted on the data representing differences in the material behavior of the specimens from middle and distal regions of the dissected aortas. A *p* < 0.05 was considered to be significant.

#### Algorithm for non-linear regression modeling

The non-linear regression techniques for determining the parameters of the HGO model were written in Python script. The data from multiple stretching protocols (1:1, 1:1.5, 1:2, 1.5:1, 2:1, with 1:1 being an equibiaxial loading condition) were used in the testing of tissues (Table 1). The following algorithm was proposed to select the best data to optimize the parameters for the HGO constitutive model:

1. Import the excel (or.csv) file that contained the load vs. displacement data recorded from the planar biaxial testing of the specimen.
2. Select the protocols (i.e., 1:1, 1:1.5, 1:2, 1.5:1, 2:1 or all of them) which were utilized in curve fitting.
3. For each of the protocols, only select the region in the recorded data that correspond to tensile loading.
4. Since the planar biaxial data may contain noise while recording, it should be filtered. We used the Locally Weighted Scatterplot Smoothing (LOWESS) algorithm (Cleveland, 1979) available in the statistical module of Python to remove noise from data.
5. After filtering the data for each protocol, we made several sets which covered data from each protocol as well as combinations of protocols. As an example, when we considered protocols (1:1, 1:1.5, 1:2, 1.5:1, 2:1), we ended up having 31 different data sets that contained 5 data sets considering each protocol, 10 data sets containing filtered data from a combination of two protocols (i.e., 1:1 and 1.5:1, 1:1 and 1:2, 2:1 and 1:1.5, etc.), 10 data sets containing filtered data from combination of three protocols (i.e., [1:1, 1:1.5, 1:2], [1:1.5, 1.5:1, 2:1], etc.), 5 data sets containing filtered data from combination of four protocols, and 1 data set containing filtered data from all protocols.
6. Using Nelder-Mead minimization algorithm (Nelder and Mead, 1965), we defined the objective function (Equation 12) considering isochoric tissue. In this function, σ_θθ_ and σ_*zz*_ were the Cauchy (true) stress data obtained from the experiments, $\sigma_{\theta \theta }^{\text{Ψ}}$ and $\sigma_{zz}^{\text{Ψ}}$ were the Cauchy stresses for the *i*^th^ point computed using Equation (9) and *n* was the number of data points. The minimization algorithm optimized the five parameters for the HGO constitutive model, namely *C*_10_, *k*_1_, *k*_2_, α, *and κ*, using the objective function given as:
  (12) $$\begin{array}{r} \chi^{2}=\overset{n}{\underset{i=1}{\sum }}\left[{\left(\sigma_{\theta \theta }-\sigma_{\theta \theta }^{\text{Ψ}}\right)}_{i}^{2}+\;{\left(\sigma_{zz}-\sigma_{zz}^{\text{Ψ}}\right)}_{i}^{2}\right] \end{array}$$
  The minimization problem is ill-conditioned and thus, has several solutions for given limits on parameters. To achieve a global minimum, the algorithm was repeated for 200 different initial values of the parameters. Only the parameter estimates corresponding to the lowest chi-square value was selected.
7. For every feasible solution we imposed conditions of *R*^2^≥0.9 and a mean square root error, ϵ ≤ 0.2 that needed to be satisfied.
8. Performing steps 1–7, we obtained parameter values for each considered combination of protocols. If the parameter values from each combination could fit the data given by the original protocols (i.e., 1:1, 1:1.5, 1:2, 1.5:1, 2:1) with a *R*^2^≥0.8 and a mean error value, ϵ ≤ 0.25, those parameter values were chosen.
9. Finally, the median of the parameter values from combinations of protocols satisfying Step 8 was computed. The median values for *C*_10_, *k*_1_, *k*_2_, *and κ* were used to plot stress-strain curves for hyperelastic tissues.
10. In case the median of the parameter values obtained from Step 9 did not fulfill the criterion laid out in Step 8, we used the parameter values for the combination that considered data points from maximum number of protocols. This combination of protocols had already fulfilled the criterion in Step 8.

The code used to compute material parameters was included as Supplementary Material, The estimated parameter values for the different regions of the dissected aortas were recorded in Tables 2–6. The data in the tables were published in Ahuja et al. (2018) and reused as part of current research.

**Table 2:** Parameter estimation for mid true lumen wall.

**Pig number**	**Thickness (mm)**	**C10 (Pa)**	***k*_1_ (Pa)**	***k*_2_**	**α (deg)**	**κ**
1	1.76	78,219	201,440	1.52	87.09	0.2
2	1.75	60,707	230,300	3.2	89.95	0.29
3	1.70	53,816	117,670	3.02	0	0.28
4	1.75	51,191	188,950	1.11	2.86	0.32
5	1.86	54,702	110,450	2.32	61.31	0.22

**Table 3:** Parameter estimation for mid false lumen wall.

**Pig number**	**Thickness (mm)**	**C10 (Pa)**	***k*_1_ (Pa)**	***k*_2_**	**α (deg)**	**κ**
1	1.3	53,456	952,380	4.94	7.45	0.3
2	1.06	88,823	94,663	18.665	0.00	0.165
3	1.04	72,082	32,735	14.9	21.20	0.11
4	1.10	19,657	45,520	2.022	49.85	0
5	1.33	53,316	53,787	6.0417	0.80	0.22

**Table 4:** Parameter estimation for mid flap.

**Pig number**	**Thickness (mm)**	**C10 (Pa)**	***k*_1_ (Pa)**	***k*_2_**	**α (degrees)**	**κ**
1	0.58	92,963	230,290	13.90	87.1	0.33
2	0.59	73,144	235,075	7.86	68.7	0.3
3	0.54	64,042	212,120	4.99	23.5	0.32
4	0.70	52,072	125,430	5.87	53.9	0.26
5	0.47	45,588	149,880	1.42	55.6	0.21

**Table 5:** Parameter estimation for distal false lumen wall.

**Pig number**	**Thickness (mm)**	**C10 (Pa)**	***k*_1_ (Pa)**	***k*_2_**	**α (degrees)**	**κ**
1	1.24	72,996	20,894	9.01	66.5	0
2	1.17	31,299	64,299	5.44	66.5	0.25
3	0.87	44,479	229,920	5.02	22.3	0.3
4	0.85	45,167	200,820	9.84	87.7	0.27
5	1.01	58,489	90,846	4.78	48.1	0.21

**Table 6:** Parameter estimation for distal flap.

**Pig number**	**Thickness (mm)**	**C10 (Pa)**	***k*_1_ (Pa)**	***k*_2_**	**α (degrees)**	**κ**
1	0.4	103,140	61,969	4.1	62.4	0.1
2	0.34	171,740	661,830	8.05	86.5	0.3
3	0.43	78,686	239,090	3.18	89.9	0.3
4	0.29	63,554	77,013	4.76	83.4	0.11
5	0.47	55,960	78,560	2.86	89.9	0.19

## Results

**Figure 3:**
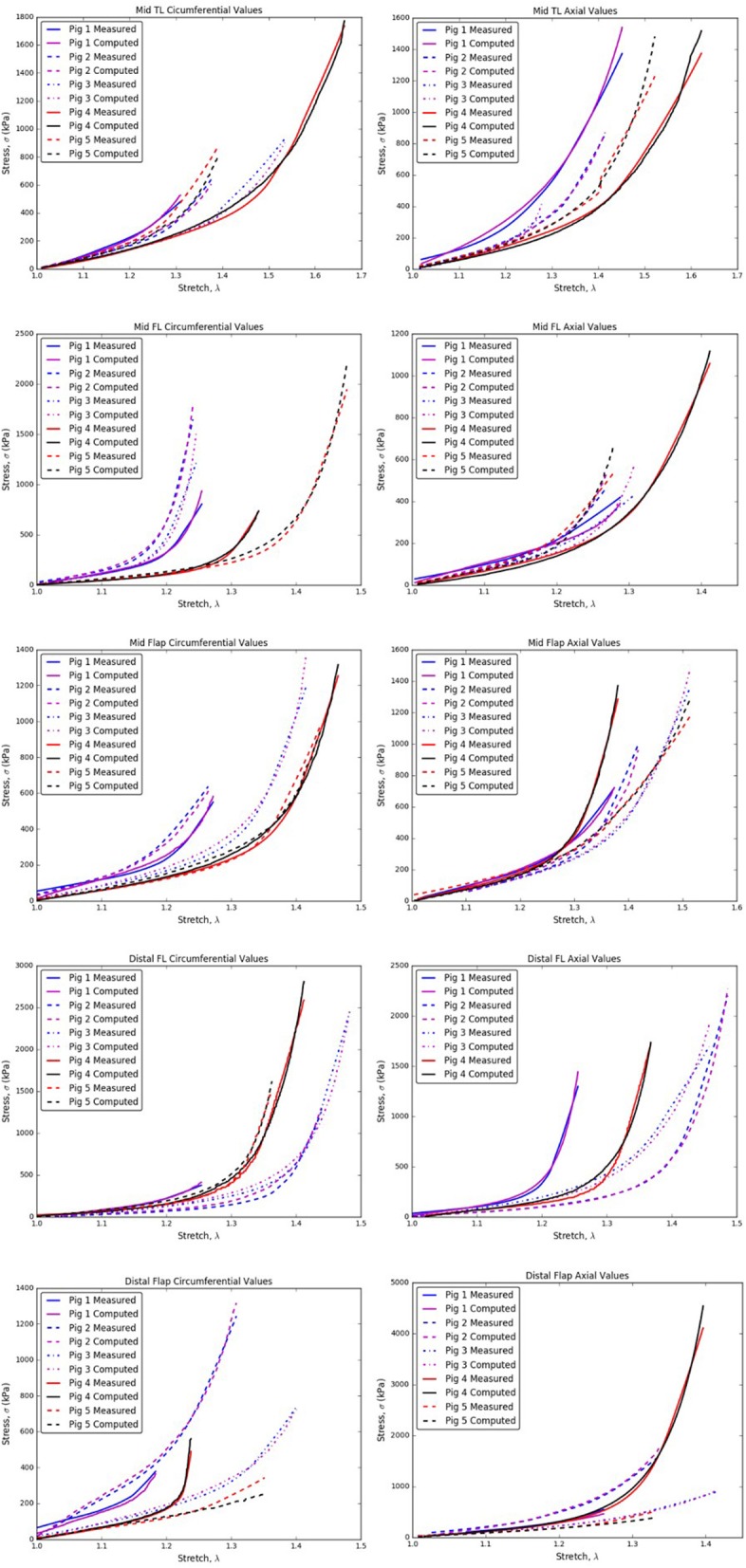
Circumferential/Axial Stress vs. Stretch relations for different regions of the dissected aorta.

The specimens and their corresponding material testing results from displacement protocols listed in Table 1 were utilized by the non-linear regression algorithm for parameter estimation. The estimation for each sample returned a *R*^2^≥0.8 and a mean error, ϵ ≤ 0.25 for every protocol that was used during the planar biaxial test measurement. The planar biaxial testing of different tissue specimens yielded the results summarized in Tables 2–6. The results in Figure 3 presented stress-stretch curves along the circumferential and axial directions for tissue samples tested with an equibiaxial (1:1) displacement-controlled protocol. It could be observed that there were differences in the mechanical response of the tissues harvested from different animals.

**Figure 4:**
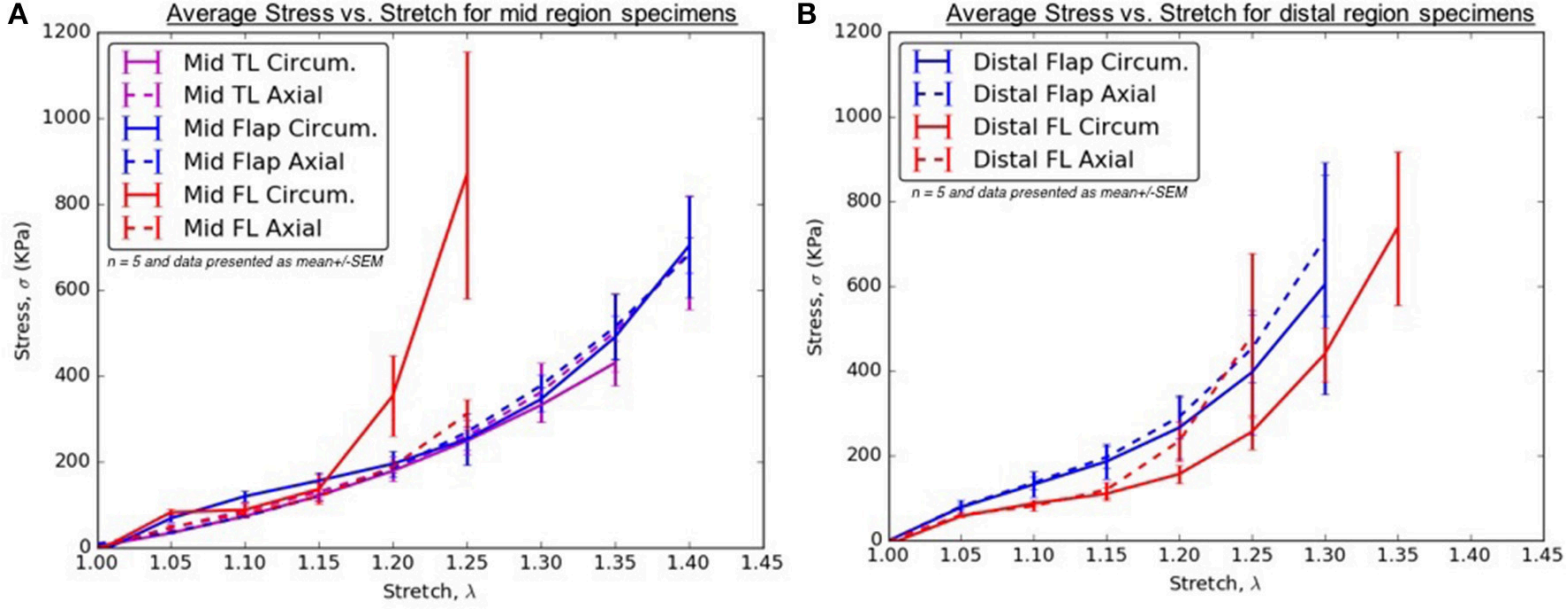
**(A)** Average Stress vs. Stretch for the different specimens harvested from the mid region **(B)** Average Stress vs. Stretch for the different specimens harvested from distal region of thoracic aorta.

**Figure 5:**
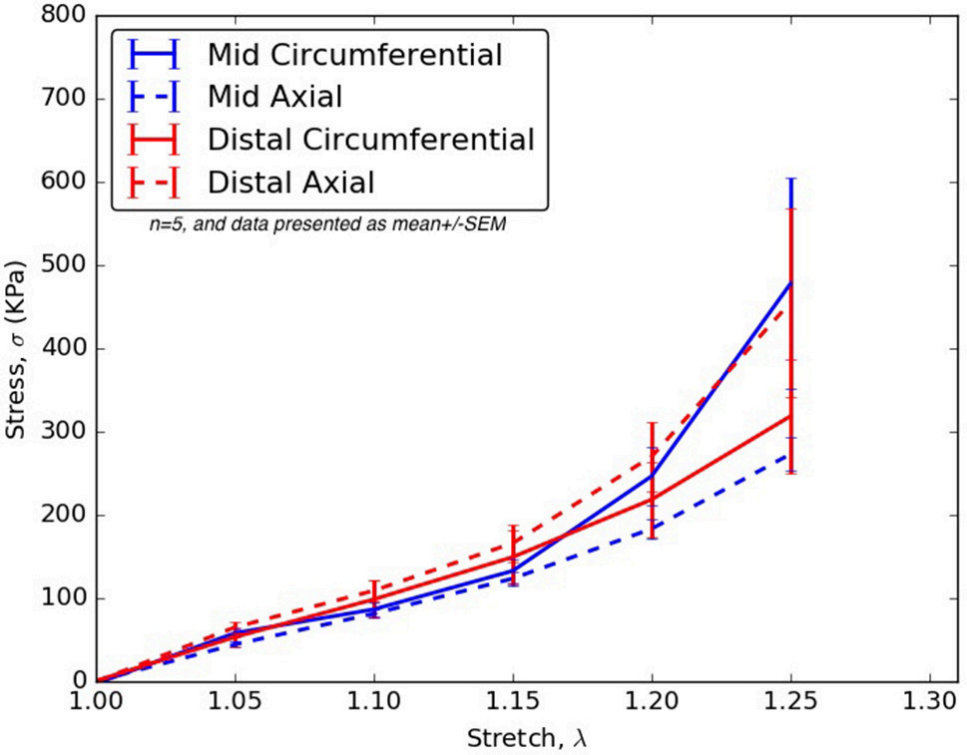
For all stretches, λ_θ_, λ_*z*_ ≤ 1.25, the axial and circumferential stresses generated in the mid and distal regions.

From Tables 2–6, the material parameters were used in Equation (9) to give the stress values for all the different stretches i.e., λ_θ_*and λ*_*z*_. The average computed results as well as the standard errors for all the different tissue specimens were presented in Figures 4, 5. Specifically in Figure 4, the variation in the stiffnesses of TL wall, FL wall, and flap harvested from the same region were compared. In Figure 5, we plotted the differences in material behaviors of specimens as one advanced from mid to distal region. The axial and circumferential stresses generated in mid and distal regions were analyzed separately using a mixed-model analyses of variance (ANOVA) using IBM SPSS Statistics (v25, IBM corporation). To accomplish it, the mid TL, mid FL, and mid flap regions were grouped into one section called “Mid” and the remaining distal regions were grouped as another section called “Distal.” For both axial and circumferential ANOVAs, mean stresses over the five stretch values (1.05, 1.10, 1.15, 1.20, 1.25) were analyzed with location (Mid vs. Distal) as the between-group variable and stretch value as the within-group (repeated measures) variable. In both axial and circumferential stress ANOVAs, the assumption of sphericity was violated and thus, the Greenhouse–Geisser correction was utilized for the stretch values.

In the ANOVA on axial stretches, results revealed a significant main effect of location [*F*_(1, 22)_ = 5.80, *p* = 0.025] with the distal region showing higher mean stresses than the mid region. There was a significant main effect of the stretch value [*F*_(1.09, 23.99)_ = 43.85, *p* < 0.001] i.e., mean stresses increased with stretch values. The location vs. stretch value interaction was not significant [*F*_(1.09, 23.99)_ = 3.24, *p* > 0.05] suggesting a similar rate of increase in stresses over the increasing stretch ratios in the mid and distal region. In the ANOVA on circumferential stretches, there was no significant main effect of location [*F*_(1, 21)_ = 0.31, *p* > 0.05], while the main effect of stretch value was significant [*F*_(1.02, 21.43)_ = 13.90, *p* = 0.001], again suggesting a similar rate of increase in stresses over the increasing stretch ratios. The location vs. stretch value interaction was not significant in the circumferential ANOVA [*F*_(1.02, 21.43)_ = 1.00, *p* > 0.05]. The comparison between mid and distal regions for axial and circumferential directions were given in Figure 5.

The variation in the material behavior of tissues were compared for a range of stretch values, 1 ≤ λ_*z*_, λ_θ_ ≤ 1.4, and the dimensionless statistic, coefficient of variation (*COV* = σ/μ, where σ is the standard deviation and μ is the mean at a specific stretch value) was calculated from computational values to indicate the extent of variability in relation to the mean. Since standard errors only reflected absolute variability among specimens, we chose *CV* to give us a relative insight into the variation in material behavior for every considered stretch value. Tables 7A–J lists the *CV* for stresses generated in tissue samples stretched to different values as well as the corresponding mean and standard deviation results. *CV* has been used to measure dispersion of critical parameter for a number of applications e.g., to measure precision and reproducibility in biological samples/assays, variability in soil compositions, etc. As there is no single *CV-*value to categorize a data series as less or more dispersive, we assumed a *COV* > 0.3 in this research as a measure of greater variation across stress data.

**Table 7A:** Statistical data for circumferential region of mid TL.

**Stretch value, λ_θ_**	***CV***
1.05	0.10
1.10	0.13
1.15	0.18
1.2	0.17
1.25	0.19
1.3	0.26
1.35	0.25

**Table 7B:** Statistical data for axial region of mid TL.

**Stretch value, λ_z_**	***CV***
1.05	0.39
1.10	0.34
1.15	0.35
1.2	0.36
1.25	0.34
1.3	0.37
1.35	0.37
1.4	0.38

**Table 7C:** Statistical data for circumferential region of mid FL.

**Stretch value, λ_θ_**	***CV***
1.05	0.36
1.10	0.38
1.15	0.46
1.2	0.66
1.25	0.73

**Table 7D:** Statistical data for axial region of mid FL.

**Stretch value, λ_z_**	***CV***
1.05	0.30
1.10	0.20
1.15	0.19
1.2	0.17
1.25	0.25

**Table 7E:** Statistical data for circumferential region of mid flap.

**Stretch value, λ_θ_**	***CV***
1.05	0.32
1.10	0.28
1.15	0.27
1.2	0.33
1.25	0.42
1.3	0.16
1.35	0.20
1.4	0.28

**Table 7F:** Statistical data for axial region of mid flap.

**Stretch value, λ_z_**	***CV***
1.05	0.12
1.10	0.09
1.15	0.10
1.2	0.12
1.25	0.11
1.3	0.15
1.35	0.30
1.4	0.11

**Table 7G:** Statistical data for circumferential region of distal FL.

**Stretch value, λ_θ_**	***CV***
1.05	0.07
1.10	0.24
1.15	0.28
1.2	0.27
1.25	0.33
1.3	0.33
1.35	0.48

**Table 7H:** Statistical data for axial region of distal FL.

**Stretch value, λ_z_**	***CV***
1.05	0.26
1.10	0.26
1.15	0.32
1.2	0.47
1.25	0.89

**Table 7I:** Statistical data for circumferential region of distal flap.

**Stretch value, λ_θ_**	***CV***
1.05	0.55
1.10	0.51
1.15	0.49
1.2	0.57
1.25	0.64
1.3	0.74

**Table 7J:** Statistical data for axial region of distal flap.

**Stretch value, λ_z_**	***CV***
1.05	0.22
1.10	0.26
1.15	0.29
1.2	0.39
1.25	0.42
1.3	0.51

**Figure 6:**
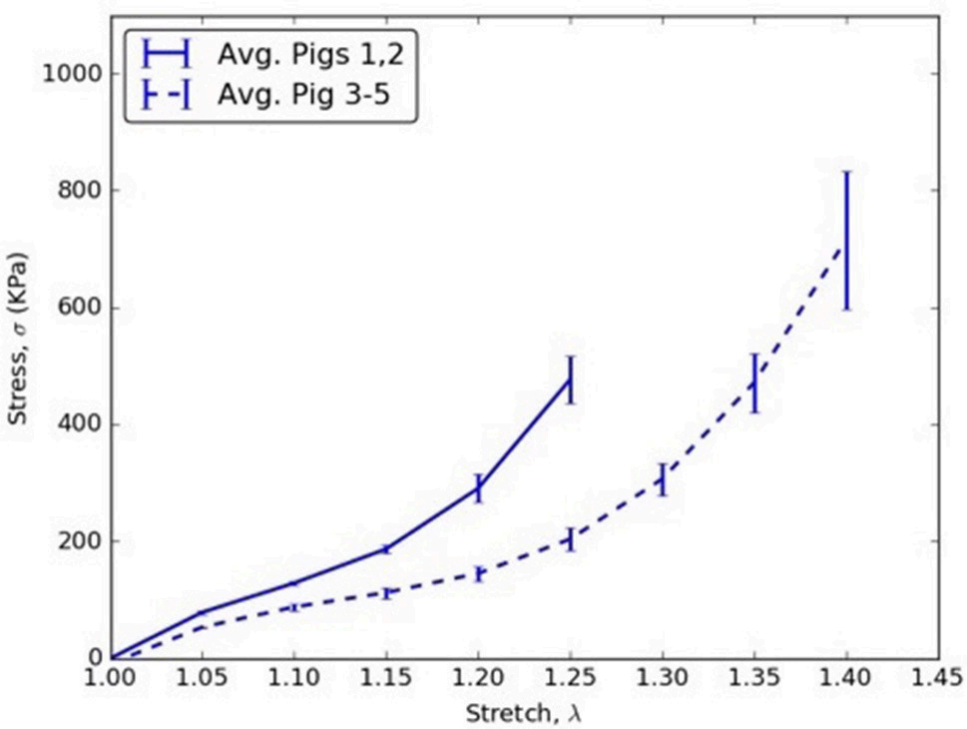
Circumferential stress vs. stretch comparison between averages of (Pigs 1 and 2) and (Pigs 3, 4, and 5) for mid flap specimens.

The following inferences were proposed based on the analysis of data in Tables 7A–J:

1. The *CVs* for mid TL wall (circumferential direction; Table 7A), mid FL wall (axial direction; Table 7D), and mid flap wall (axial direction; Table 7F) were < 0.3 for all stretch values. Thus, there was less variation in tissue data for all stretches.
2. In the axial direction, the *CVs* for mid TL wall was >0.3 for all stretches, indicating dispersion in data. A large variation was observed between data from pig 1 and data from all other pigs (Table 7B and Figure 3).
3. In mid FL wall (Table 7C) and distal flap (Table 7I), the stresses along circumferential direction led to *CV-*values >0.3. We observed a greater dispersion behavior between all specimens.
4. The circumferential direction of mid flap (Table 7E) resulted in *CVs* >0.3. The variation is shown in Figure 6 and is attributed to differences between (Pigs 1 and 2) and (Pigs 3, 4, and 5).
5. The data analysis on circumferential directions for distal FL (Table 7G) yielded *CVs* > 0.3 only for stretches, λ_θ_≥1.25. Hence, at higher stretch values, there was variation among tissue specimens. In terms of mathematical formulation, Ψ_*aniso*_ dictated the differences in the distribution of collagen fibers which led to variation in the stress responses at higher stretch values.
6. Similarly, stretching distal flap along axial direction (Table 7J) resulted in *CV-*values > 0.3 for stretch values, λ_*z*_≥1.20.

## Discussion

AD is the most common life-threatening disorder affecting the aorta. The literature is replete with material behavior characterization of thoracic and abdominal aortic aneurysm wall (Raghavan and Vorp, 2000; Thubrikar et al., 2001; Vorp, 2007; Speelman et al., 2009) but the data on mechanics of dissected aortic wall and intimal flap is incomplete (Pasta et al., 2012). A better understanding of this critical condition is warranted since greater number of patients are undergoing endovascular treatments which requires interaction between the walls, the intimal flap of the dissection and endograft, or bare metal stent. This will allow us to assess the design and long-term utilization of aortic grafts. Thus, characterizing the material response of the different regions of dissected aorta using a structure based form of strain energy function will be useful in constructing well-informed computational models (e.g., FEA and FSI) which will expedite the development of endovascular therapy for successfully sealing the FL and thereby reducing mortality and future reinterventions.

### Non-linear regression analysis for constitutive modeling

The present study created artificial dissections in porcine aortas, conducted planar biaxial testing on tissue segments from different regions and then used a novel non-linear regression modeling interface to fit the five parameter HGO model for hyperelastic materials against measured data. The constitutive modeling for soft tissues has been widely utilized to understand its mechanical response and perform computational modeling for developing virtual therapies for treating diseases (Raghavan and Vorp, 2000; Holzapfel et al., 2004; Speelman et al., 2009; Patel et al., 2018). The algorithm introduced in this study utilized data from various protocols (i.e., 1:1, 1:1.5, 1:2, 1.5:1, 2:1) to develop an enriched HGO constitutive model relation that was trained on larger data set for achieving higher stress-stretch predictive capabilities (Table 1). The algorithm was validated by ensuring that the parameters selected for the HGO constitutive model could fit the measured data from each protocol with a *R*^2^≥0.8 and a mean error value of ϵ ≤ 0.25. This was in contrast with the approaches followed traditionally in literature (Zeinali-Davarani et al., 2013; Babu et al., 2015), where data collected from only equibiaxial protocol was considered or parameters were estimated from entire data set (Billiar and Sacks, 2000) without validating the constitutive model response to new data. This study proposed a rigorous algorithm for considering multiple combinations of material testing protocols. The resultant parameters estimated from different combinations were pooled together and the median values for *C*_10_, *k*_1_, *k*_2_, α, *and κ* were computed. Only in the case when median values did not fit the data (Step 8 in section Algorithm for Non-linear Regression Modeling), the parameter values for the combination that considered data points from maximum number of protocols were used. The implementation of this novel algorithm was undertaken to propose a well-informed constitutive model that would allow development of a better computational model for understanding the mechanics of the aortic tissue in health and disease.

The material parameters, *C*_10_, *k*_1_, *k*_2_, α, *and κ*, for all tissue specimens from different regions of the dissected aorta were presented in Tables 2–6. These material parameters were used, as it is, for developing specimen-specific computational models for reproducing AD and analyzing the effects of therapy on the treatment of the disease (Ahuja et al., 2018).

### Clinical relevance

A healthy aorta is pre-stretched axially to carry the pulse pressure with minimal variation in its length (Van Loon, 1977; Schulze-Bauer and Holzapfel, 2003; Sommer and Holzapfel, 2012). In the circumferential direction, the aorta resists distensibility by stiffening at higher stretches. With the creation of FL due to AD, two new regions, namely FL wall and intimal flap are created. It becomes important to highlight the importance of material response with respect to the circumferential and axial directions to support therapy as well as predict potential complications.

Patients with AD suffer from hypertension, which significantly adds to the existing longitudinal stresses in aorta leading to circumferential tearing along this orientation. As the dissected aorta dilates, the circumferential stresses on mid and distal FL wall increases according to Laplace's law. The weakened wall of distal FL has higher propensity to dilate at lower stresses along circumferential direction (Figure 4B) and as a result, there is a risk of aneurysm formation in patients (Lopera et al., 2003; Won et al., 2006). Furthermore, the blood flow induces additional normal and shear stresses on the compromised distal FL wall. Future simulations utilizing region-specific material properties would be required to understand the relationship between hemodynamics and structural loading and the formation of aneurysms in distal FL walls.

For treatment of AD, an endograft is first deployed to exclude the proximal entry tear to redirect blood flow toward the TL and then a stent graft is used to push the intimal flap against the FL wall such that the aorta is reconstituted by sealing the FL. The deployment of stents/graft will be dependent on circumferential stiffnesses of mid and distal flaps. According to our results in Figure 5, significant differences were not observed in circumferential stresses between mid and distal regions. This observation will be an important factor in sizing stents (Ahuja et al., 2018).

### Stiffness of TL wall, FL wall, and flap harvested from the same region

In the mid region, the stress-stretch plots for each region were superimposed and shown in Figure 4A. It was observed that the mid FL region was the stiffest when tested biaxially in both the circumferential and axial directions. A slightly higher stiffness was observed along the circumferential direction in the mid region of the flap for small stretch values (λ_θ_ < 1.2). At higher stretch values, the curves for mid region of flap overlapped indicating similar material behavior along the two principal directions. Lower stress values in the circumferential direction of the mid TL region indicated higher density of collagen fibers along axial direction.

In the distal region, a different trend was observed for the flap and the FL wall. The flap tissue was stiffest in the axial direction as shown by high stresses in Figure 4B. On the contrary, the FL wall was the least stiff along the circumferential direction but eventually became stiffer and showed asymptotic material behavior in the axial direction for λ_*z*_>1.2.

### Differences between mid and distal regions

The results in Figure 5 showed that the mid region tissue was stiffer along circumferential direction at higher stretches (λ_θ_ ≥ 1.2) which decreased as one advanced toward the distal region. The axial direction was stiffer in the distal region suggesting greater presence of collagen fibers along that direction. Thus, a change in the distribution and orientation of the collagen fibers as one moved from mid to distal region of dissected aorta was observed.

### Variability between tissue specimens from different pigs

The results showed that CVs > 0.3 were obtained for all studied tissue regions (i.e., mid TL, mid FL, mid flap, distal FL, distal flap). In all tissue regions except mid TL, the CV-values were larger along circumferential direction as compared to axial direction. This could be attributed to the differences in the collagen fiber content and their orientations, which led to higher variability between specimens. Consequentially, it becomes imperative to perform patient-specific measurements and computations for choosing the accurate therapy to treat AD patients.

## Limitations of study

The planar biaxial testing methodology assumed aortic tissue samples as incompressible because of the presence of high water content. As a result, the stretch of tissue along the radial direction was given as λ_*r*_ = 1/λ_1_λ_2_. A small error was introduced in our calculations because of this assumption (Taghizadeh et al., 2015). Moreover, the experimental studies conducted for this research did not include the effects of in-plane shear as we assumed the x- and y-directions for the specimens to be oriented along the principal directions; i.e., circumferential and axial. A method proposed in Sacks (2000) oriented specimens at specific angles to produce a state of simultaneous in-plane shear and normal strains. This method would be explored and incorporated in future studies if the in-plane shear strains are comparable to the normal strains. Our current approach optimized the five parameters for the HGO constitutive model, namely *C*_10_, *k*_1_, *k*_2_, α, and κ, using an objective function. The orientation and distribution of fibers in the tissue specimens, represented by α and κ, respectively, were calculated numerically. An alternative approach would be to conduct histology on the dissected aorta specimens to visualize the collagen fibers using fluorescence microscope. The histological measurements of α and κ can then be incorporated into the HGO constitutive model for optimizing the remaining parameters, *C*_10_, *k*_1_, *k*_2_. Even though the biaxial measurements were conducted on tissue specimens within 16 h of harvesting, there is a concern regarding swelling of these samples. In future, biaxial tests would be undertaken on fresh samples and compared with samples that have been stored over certain number of hours. This would allow in precisely predicting the enzymatic degradation of tissue samples over a range of time span.

Further, it was realized that the use of healthy tissues could be a limitation in comparing with an actual dissection where the aortic wall is diseased and weak. The presented mathematical model was developed to compare with our developed acute *in-vivo* porcine animal model which is out of the scope of current paper. Nonetheless, these results provided insight into type B dissections occurring, for example, as a result of blunt chest trauma from motor accidents (Turhan et al., 2004).

## Conclusions

From the results presented above, it was shown that there were significant differences in the mechanical responses of tissue specimens harvested from different regions of a dissected aorta. Hence, the null hypothesis was true, and it was suggested that one should use region-specific material properties when simulating the structural and hemodynamic response of a dissected aorta to external loading. In future, accurate simulations would allow in advancing the development of properly sized grafts for treating AD and thereby, reducing patient reinterventions during followups.

## Ethics statement

The aortas required for conducting experiments were sourced from a service oriented company (Sierra for medical science, Whittier, CA) that provides biological tissues to the consumers for research and development. The company requests animal tissues postmortem from only USDA-approved slaughterhouses throughout the nation. Hence, ethical approval was not requested for current study.

## Author contributions

AA is the first author of this research and contributed to this manuscript extensively. JN prepared the samples and supervised the bench testing. TT conducted bench testing and saved the data in.csv files. BP assisted AA with the biomechanical characterization of tissues. JK provided expert insight into the material behavior of tissues and assisted with the calibration and functioning of bench testing machine. BP, JN, SC, and GK contributed to critical sections of the paper, the protocol, and revision of the drafts.

### Conflict of interest statement

Study is funded by 3DT Holdings and Cook Medical Inc. The employees of the funding company Cook medical participated in the study and are included in the author list. GK has received funding for this project from Cook Medical Inc. The author list contains representatives from Cook Medical Inc. that includes JN, JK, BR, and SC. The authors declare that the research was conducted in the absence of any commercial or financial relationships that could be construed as a potential conflict of interest.

## Footnotes

^1^1:1 displacement protocol is also referred to as equibiaxial displacement protocol.
